# Translational regulation contributes to the elevated CO_2_ response in two *Solanum* species

**DOI:** 10.1111/tpj.14632

**Published:** 2020-01-16

**Authors:** Sharon B. Gray, Joel Rodriguez‐Medina, Samuel Rusoff, Ted W. Toal, Kaisa Kajala, Daniel E. Runcie, Siobhan M. Brady

**Affiliations:** ^1^ Department of Plant Biology and Genome Center University of California, Davis 451 Health Sciences Drive Davis CA 95616 USA; ^2^ Department of Plant Sciences University of California, Davis One Shields Avenue Davis CA 95616 USA; ^3^ Present address: Plant Ecophysiology Utrecht University Padualaan 8 3584 CH Utrecht the Netherlands

**Keywords:** elevated carbon dioxide, *Solanum*, genetic variation, root, transcriptome, translatome, metabolome, regulation

## Abstract

Understanding the impact of elevated CO_2_ (eCO_2_) in global agriculture is important given climate change projections. Breeding climate‐resilient crops depends on genetic variation within naturally varying populations. The effect of genetic variation in response to eCO_2_ is poorly understood, especially in crop species. We describe the different ways in which *Solanum lycopersicum* and its wild relative *S. pennellii* respond to eCO_2_, from cell anatomy, to the transcriptome, and metabolome. We further validate the importance of translational regulation as a potential mechanism for plants to adaptively respond to rising levels of atmospheric CO_2_.

## Significance Statement

In a domesticated and a wild *Solanum* species, translational and transcriptional regulation feedback onto each other and show interspecific differences in response to elevated CO_2_.

## Introduction

Atmospheric CO_2_ has increased more than 40% since pre‐industrial times and a similar magnitude of increase is predicted by the end of this century (IPCC, 2013). Plants are autotrophic and their prime substrate for photosynthetic production of sugars is CO_2_. In C_3_ species, photosynthetic CO_2_ assimilation is limited by the diffusion of CO_2_ from the atmosphere into the chloroplast and the biochemical capacity for CO_2_ fixation in the chloroplast. Consequently, growth at elevated CO_2_ concentration (eCO_2_) generally stimulates photosynthetic CO_2_ assimilation, which in turn leads to greater leaf respiration and plant growth when sufficient nutrients and water are available (Drake *et al.*, 1997; Ainsworth and Long, 2004; Leakey *et al.*, 2009a,b).

Meta‐analyses of C_3_ species investigated at 12 Free Air CO_2_ Enrichment (FACE) facilities with long‐term application of eCO_2_ supports this general biomass stimulation with an average increase in above‐ground biomass of 20% in response to eCO_2_. Yet, different plant species respond in different ways to CO_2_ (Bishop *et al.*, 2014). Presumably, this diversity of responses involves complex interactions among plant carbon, water, and nutrient relations, which are also constrained by plant developmental programmes and gene regulation (Leakey and Lau, 2012; Gray and Brady, 2016). As a consequence, improving plant performance under future growing conditions requires a global understanding of both the physiological, developmental and regulatory mechanisms that govern the response to eCO_2_ across organs and cell types in diverse plant species. Capturing genetic variation associated with enhanced yield under eCO_2_ would be highly valuable for breeding crop varieties for the future.

The output of genetic variation is phenotypic variation in the form of physiological, developmental, cellular, and molecular traits. The physiological traits most directly impacted by elevated CO_2_, with significant subsequent impacts on growth and yield, are photosynthetic carbon assimilation and stomatal conductance (Ainsworth and Long, 2004). Much of the previous research investigating the effects of eCO_2_ on plant development has focused on above‐ground tissues (Taylor, 2003; Dermody *et al.*, 2006; Gray and Brady, 2016). However, because yield responses to eCO_2_ ultimately depend on nutrient and water status as well as sink‐source balance for carbon, root function is recognized to be very important for plant responses to eCO_2_ (Rogers *et al.*, 1994; Zak *et al.*, 2000; Gray *et al.*, 2012). Nevertheless, knowledge of how root development is altered in response to eCO_2_ lags far behind our understanding of shoot responses.

Stimulatory effects of eCO_2_ on root biomass have been observed in several plant species, including Arabidopsis (Crookshanks *et al.*, 1998; Jauregui *et al.*, 2015), wheat (Vicente *et al.*, 2016), and tomato (Cohen *et al.*, 2018). Whether or not genes that determine root system architecture do so in a Mendelian manner in response to eCO_2_ across plant species is less clear. At the cellular level, changes in root and shoot function can occur through modification of xylem cell development or hydraulic conductivity. As the majority of water acquired by roots is transported from roots to shoots before diffusing into the atmosphere via stomata in exchange for CO_2_, understanding xylem vessel diameter is also critically important to understanding plant water relations. Root xylem vessel diameter was shown to increase in tomato (cv Ikram) in response to eCO_2_ in different concentrations of nitrate (Cohen *et al.*, 2018). These studies demonstrate that root cellular anatomy should be an additional phenotypic trait used to quantify genetic variation in a plant's response to eCO_2_.

Molecular mechanisms underlie these morphological and physiological responses. Monitoring the transcriptome in leaves has revealed gene expression patterns that closely mirror changes in metabolism (Kaplan *et al.*, 2012; Watanabe *et al.*, 2014; Markelz *et al.*, 2014a; Jauregui *et al.*, 2015; Vicente *et al.*, 2016; Zhang *et al.*, 2018; Vicente *et al.*, 2018). In soybean, eCO_2_ also induced significant changes in the expression of genes associated with carbohydrate metabolism and respiration (Leakey *et al.*, 2009a,b). In Arabidopsis, eCO_2_ induced expression of genes encoding respiratory machinery, components of the glycolytic cycle, the tri‐carboxylic acid (TCA) cycle, and the mitochondrial electron transport chain, which is consistent with increased leaf respiration and foliar starch and sugar content (Leakey *et al.*, 2009a,b). The root transcriptome of Arabidopsis in response to eCO_2_ has recently been characterized and indicates potential mechanisms for differences in organ‐level N assimilation (Jauregui *et al.*, 2016).

In cereal crops, grain protein content is reduced in response to elevated carbon dioxide (Medek *et al.*, 2017). Despite these organ‐specific changes in protein level, to the best of our knowledge, translational regulation in response to eCO_2_ has not been analyzed previously. The association of specific transcripts with ribosomes is dynamic, and changes spatiotemporally (Mustroph *et al.*, 2009; Lin *et al.*, 2014; Tian *et al.*, 2019) as well as in response to many abiotic stresses including light (Juntawong and Bailey‐Serres, 2012; Liu *et al.*, 2013), heat (Yángüez *et al.*, 2013; Lukoszek *et al.*, 2016), salt (Li *et al.*, 2018), hypoxia (Mustroph *et al.*, 2009), dehydration (Kawaguchi *et al.*, 2004), and phosphate deficiency (Bazin *et al.*, 2017). In these cases, transcripts associated with primary metabolism and translation often show reduced ribosomal association, while genes associated with the stress response show increased ribosomal association. These changes are thought to be adaptive and present an additional regulatory mechanism by which a plant responds to stress.

Given the importance of identifying genetic variation to breed plants with an improved response to eCO_2_, we characterized the eCO_2_ response of the domesticated tomato species, *Solanum lycopersicum*, and its wild relative, *S. pennellii*. *S. pennellii* is a desert‐adapted species, that displays salt and drought stress tolerance (Dehan and Tal, 1978; Koca *et al.*, 2006; Easlon and Richards, 2009). Furthermore, these two species have striking differences in root anatomy, morphology, and gene expression; these are likely to contribute to their differences in response to stress (Ron *et al.*, 2013; Toal *et al.*, 2018) and provide a compelling opportunity to assess how closely related species with significant differences in root morphology and anatomy respond differentially to eCO_2_. We used *S. lycopersicum* and *S. pennellii* to address the following question: Which physiological, cell anatomical and molecular mechanisms are different between a crop species and a wild drought‐tolerant relative in response to eCO_2_? While extensive genetic variation in a range of stress responses has been observed between these two species (Gong *et al.*, 2010; Frary *et al.*, 2010; Frary *et al.*, 2011), they respond in a similar physiological manner in response to eCO_2_. Specific differences in how these species respond include changes in xylem vessel anatomy, a small number of metabolites, and transcriptional regulation, particularly in genes associated with translation. We determined that these transcriptional differences lead to changes in translation, and present a newly described mode of regulation in response to eCO_2_, that is, differential polysome occupancy of transcripts. Furthermore, this translational regulation serves to dampen transcriptional regulation. These results have implications for improving our basic understanding of species‐specific responses to eCO_2_ and the genetic variation available for breeding climate change‐resilient crops.

## Results and Discussion

### Elevated CO_2_ increases photosynthesis and biomass in both *S. lycopersicum *and *S. pennellii*

**Figure 1:**
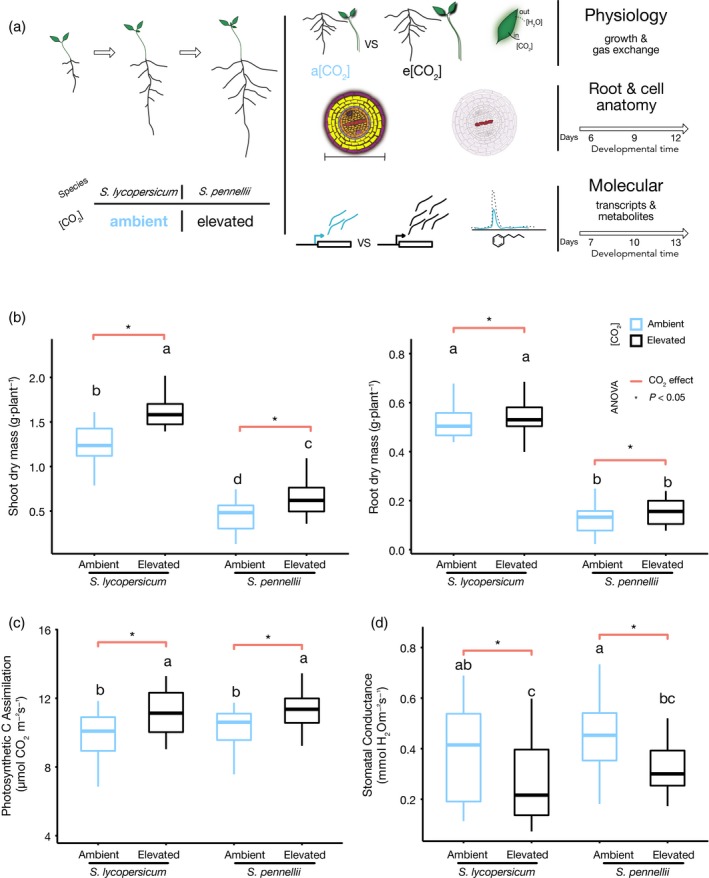
Experimental design and *Solanum lycopersicum/S. pennellii* physiological and growth responses to eCO_2_. (a) Experimental design to characterize the interspecific response to elevated CO_2_ of *S. lycopersicum* and *S. pennellii.* Root system architecture, cell anatomy, and physiological measurements were recorded. Cell anatomy measurements were sampled at 6, 9 and 12 days after planting (DAP). Molecular data included metabolites and transcriptional profiles sampled at 7, 10 and 13 DAP (right side). (b–d) Blue = ambient CO_2_; black = elevated CO_2_. (b) Elevated CO_2_ has a significant (*P* < 0.05) effect in shoots (left panel) and roots (right panel) for dry mass in both species (two‐way anova). (c) Photosynthetic rate is increased in response to elevated CO_2_ (two‐way anova). (d) Stomatal conductance decreased significantly due to elevated CO_2_ (two‐way anova). The letters above the boxplots are the results of a Tukey honest significant difference (HSD) test. Orange lines above boxplots indicate if there was a significant effect of CO_2_ (anova, *P* < 0.05; Table S1).

We measured changes in root and shoot biomass, the photosynthetic rate of leaves, and stomatal conductance under ambient and eCO_2_ in *S. lycopersicum* cv M82 (written as *S. lycopersicum* throughout) and *S. pennellii* at 7, 10, and 13 days after planting (DAP) (Figure 1a). It should be noted that differences in plant morphology between these species do not represent a developmental delay (Ron *et al.*, 2013; Koenig *et al.*, 2013). Samples were collected at equivalent times of the day, although this does not preclude small differences in developmental timing between samples. We observed a significant net increase of dry biomass in shoots (*F*_1,92_ = 45.8, *P* < 0.05) and roots (*F*_1,92_ = 4.5, *P* < 0.05) in both species in response to eCO_2_ (Figure 1; Table S1). The photosynthetic carbon assimilation rate increased significantly in response to eCO_2_ (*F*_1,92_ = 24.5, *P* < 0.05), consistent with other studies (Markelz *et al.*, 2014b; Gray and Brady, 2016) (Figure 1c and Table S1). Stomatal conductance had a significant net decrease (*F*_1,92_ = 23.9, *P* < 0.05) (Figure 1d and Table S1), also as previously reported in other species (Hussain *et al.*, 2013; Gray and Brady, 2016). There was no difference in how these two species responded to eCO_2_ for dry biomass of shoots and roots, photosynthetic carbon assimilation rate and stomatal conductance, as determined by the lack of a significant interaction effect (*P* > 0.05) between species and CO_2_. Thus, these two species respond in a similar physiological manner to other C3 plant species: an increase in plant biomass in response to eCO_2_ due to an increase in photosynthesis and decrease in stomatal conductance (Crookshanks *et al.*, 1998; Markelz *et al.*, 2014a), but there is no variation in their response.

### Elevated CO_2_ alters root cell anatomy in *Solanum* species

**Figure 2:**
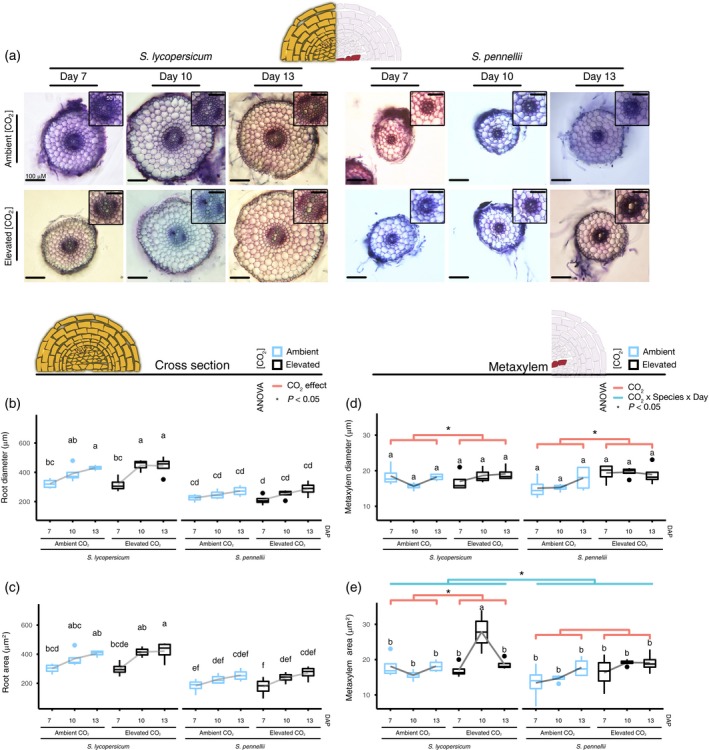
Metaxylem development changes in different ways between species in response to eCO_2_. (a) Cross‐sections taken at 1 cm from the root tip of *Solanum lycopersicum* (left half) or *S. pennellii* (right half) at 7, 10, or 13 days after planting (DAP), under ambient (upper row) or elevated (bottom row) CO_2_. Inset = vascular area. Scale bars (main figure) = 100 μm (0.1 mm); scale bars (inset) = 50 μm (0.05 mm). A 3‐way anova revealed no significant effect of [CO_2_] on root diameter (b) or area (c). Conversely, metaxylem diameter (d) and area (e) were affected by [CO_2_]. Letters above the boxplots are the results of a Tukey HSD test. Lines above boxplots indicate if there was a significant effect of CO_2_ (orange) or an interaction (CO_2_ × Species × Day in blue) effect (anova, *P* < 0.05; Table S2).

Although these species respond in a similar physiological manner, it is possible that they do so through the execution of different regulatory pathways. This hypothesis is further supported by the existing differences in development between *S. lycopersicum* and *S. pennellii*. Root biomass changes in a very minimal manner in response to eCO_2_. Thus, we chose to only characterize root anatomical changes. We compared the area and diameter of the root radial axis and metaxylem in response to eCO_2_ treatment (700 ppm versus 400 ppm for ambient conditions) for 7, 10, and 13 DAP (Figure 2a). There was no difference in these traits for either species (Figure 2b,c and Table S2) in the root radial axis. In contrast, we found a difference in two aspects of metaxylem development in response to eCO_2_; a net increase in the developing metaxylem area (*F*_1,35_ = 11.35, *P* < 0.05) and diameter (*F*_1,35_ = 8.3, *P* < 0.05) (Figure 2d,e and Table S2). The way in which metaxylem area changes differed between species. Metaxylem area was much larger and had more variation in *S. lycopersicum* at 10 DAP relative to *S. pennellii* (Figures 2e and S1; there was a significant interaction effect between species and developmental stage in response to eCO_2_; *F*_2,35_ = 3.6, *P* < 0.05). Furthermore, this change in metaxylem area was not due to the change in root diameter or area (Figure S1 and Table S2). This contrasts with our photosynthesis and biomass measurements for which both *Solanum* species responded in a similar manner. The metaxylem differences between species provide support that a different mode of regulation in the eCO_2_ response might exist between both species.

To further understand the effect of eCO_2_ in the xylem of mature plants, we measured xylem vessel area and vessel number in 12‐week‐old plants; however, these traits were no longer eCO_2_ responsive (Figure S2 and Table S2). Taken together, these results point to a targeted effect of eCO_2_ in the root that is specific to the early developing metaxylem. The eCO_2_‐dependent increase of the metaxylem area could increase hydraulic conductivity and enable the increase in shoot biomass.

### Elevated CO_2_ perturbs the accumulation of a subset of metabolites

**Figure 3:**
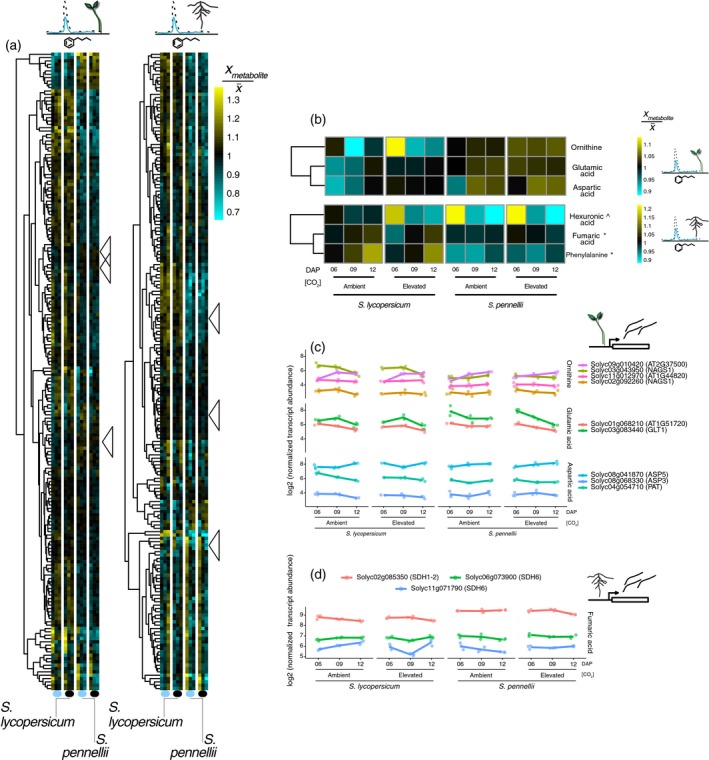
Elevated CO_2_‐responsive metabolites and associated pathways in shoots and roots. (a) Heatmap of the 191 metabolites profiled. Colour scale represents the mean‐normalized ratio of the log_2_ metabolite concentration. Significant metabolites with a CO_2_ (main or interaction) effect (*q*‐value <0.05) are marked with arrowheads: glutamic acid, ornithine and aspartic acid in shoots (b, upper panel); in the roots, a CO_2_‐related effect was significant for phenylalanine, fumaric acid, and hexuronic acid (b, lower panel. Superscript indicates the type of interaction term: ^CO_2_ × Day; *CO_2_ × Species). Mean‐normalized transcript abundance from total RNA‐seq of selected genes in shoots (c) and roots (d) related to the metabolite synthetic pathways of differentially accumulated metabolites.

Elevated CO_2_ is known to target primary metabolism. To address potential changes between species in metabolite accumulation in response to eCO_2_ in both species, we profiled primary metabolites in the shoots and roots of *S. lycopersicum* and *S. pennellii* at three time points 6, 9, and 12 DAP; staggered from the anatomical measurements for ease of sampling (Figure 3a). Our analysis identified 180 metabolites in the roots and 140 metabolites in the shoots whose concentration changed significantly (*q*‐value <0.05; Table S3) in a species, day, or CO_2_‐dependent manner (either as a main effect or interaction). Only three out of the 140 metabolites that changed in the shoots, changed in response to eCO_2_: glutamic acid (CO_2_; logFC = 0.97, *q*Val < 0.05), ornithine (CO_2_; logFC = 1.44, *q*Val < 0.05), and aspartic acid (CO_2_; logFC = 1.56, *q*Val < 0.05) (Figure 3a,b and Table S3). Three of the 180 differentially accumulated root metabolites changed their abundance in response to eCO_2_, two of which changed in distinct ways between the two species, as well as in developmental time (Figure 3b and Table S3): fumaric acid (CO_2_ × Species × Day 12; logFC = −1.11, *q*Val < 0.05) and phenylalanine (CO_2_ × Species; logFC = 0.69, *q*Val < 0.05). Hexuronic acid (CO_2_ × Day 12; logFC = −2.44, *q*Val < 0.05) abundance changed in response to eCO_2_ in a similar way in both species. These results provided further evidence of interspecies variation in response to eCO_2_ at the molecular level. The observed changes in accumulation of glutamic acid and ornithine in response to eCO_2_ are similar to previous observations in Arabidopsis, in which the metabolites changed in response to eCO_2_ but not between ecotypes (Li *et al.*, 2008a). No previous studies have reported changes of individual metabolites whose response to eCO_2_ varied between genotypes or species.

### Distinct CO_2_‐dependent transcriptional reprogramming of *S. lycopersicum* and *S. pennellii*

To link the molecular mechanisms underlying our observed physiological, developmental, and metabolic plant responses to eCO_2_, we analyzed the transcriptomic landscape of shoots and roots in both species over three developmental time points (6, 9, and 12 DAP). We explored the transcript accumulation of genes involved in glutamate to ornithine biosynthesis, 2‐oxoglutarate to glutamic acid (l‐glutamate) biosynthesis, aspartic acid biosynthesis, and succinate to fumarate biosynthesis to determine if transcriptional regulation of metabolic enzymes is responsible for the differential accumulation of metabolites. The transcript accumulation patterns of these enzymes revealed no discernible pattern of transcript accumulation in relation to metabolite concentration for either species in response to eCO_2_ (Figure 3c,d). Therefore, differential accumulation of these metabolites is likely to not be transcriptionally regulated.

**Figure 4:**
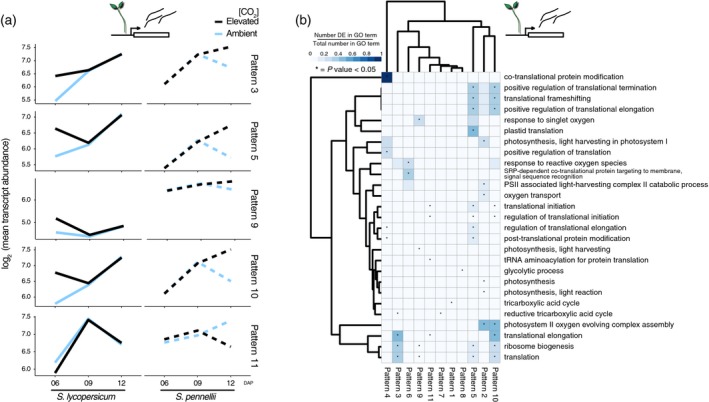
Elevated CO_2_ perturbs regulatory modules associated with translation. (a) Representative line plots of gene expression patterns associated with translation. Lines represent the average log_2_ normalized transcript abundance (total RNA‐seq) of all genes assigned to a dominant pattern; blue = ambient CO_2_; black = elevated CO_2_. (b) Enriched GO categories associated with dominant patterns. Scale = ratio between the number of significant genes in the category and the total number of genes assigned to the same category (*P < 0.05).

Our results suggested that, although *S. lycopersicum and S. pennellii* have similar physiological responses to eCO_2_ (increase of photosynthesis and biomass with a decrease in stomatal conductance), the root anatomic and metabolomic data provided evidence that distinct biological programmes can be employed to give rise to these similarities. We therefore hypothesized that the underlying transcriptional regulatory network coordinating the eCO_2_ response may be different between the *Solanum* species. This hypothesis was supported by analyzing whole transcriptome expression patterns. The two species primarily diverged in their eCO_2_‐responsive transcript accumulation at 12 DAP (Figure S3a,b). Clustering of these divergently expressed genes between species combined with Gene Ontology (GO) enrichment analysis revealed the putative functions of these genes (Figure 4a,b). In the root, four clusters (patterns 1, 2, 3, and 7) were associated with ribosome biogenesis and translation; other clusters were associated with the plasma or vacuolar membrane, GTP‐associated signaling, and the cell wall (Figure S4b–d and Table S4). As anticipated (Watanabe *et al.*, 2014; Markelz *et al.*, 2014a; Jauregui *et al.*, 2015), shoot genes associated with glycolysis and the TCA cycle were enriched, in addition to photosynthesis (Figure 4b). Surprisingly, genes associated with translation or ribosome biogenesis (Figure 4b) were significantly differentially expressed in five co‐expressed gene groups (patterns 3, 5, 9, 10, and 11; Figure 4a). These processes differed slightly between the root and shoot; processes associated with ribosome protein subunit biogenesis were enriched in the root (Figure S4d), while processes associated with translational initiation and elongation were enriched in the shoot (Figure 4b). We further analyzed the promoters of genes assigned to these dominant patterns (3, 5, 9, 10, and 11) in shoots. We found 41 significantly enriched motifs (adjusted *P*‐value <0.01, Bonferroni correction; Table S4) that represent potential transcription factor binding sites common to all patterns (Figure S4e). The TFIIIA binding motif was highly enriched in patterns 3, 5, 10, and 11. TFIIIA is specifically required for the transcription of 5S ribosomal RNA, an important component of the ribosome (Layat *et al.*, 2013). Overexpression of TFIIIA in rice enhanced its tolerance to drought, cold, and salt stresses (Huang *et al.*, 2012).

Divergent transcriptional changes between species in response to eCO_2_ have not been previously reported, neither have changes in translation. We thus hypothesized that there may be changes in which transcripts are associated with ribosomes in the shoot in response to eCO_2_. These transcriptional changes represent averaged changes across organs and we anticipate an increased complexity of changes within and across individual cell types.

### Preferential transcript loading on ribosomes

**Figure 5:**
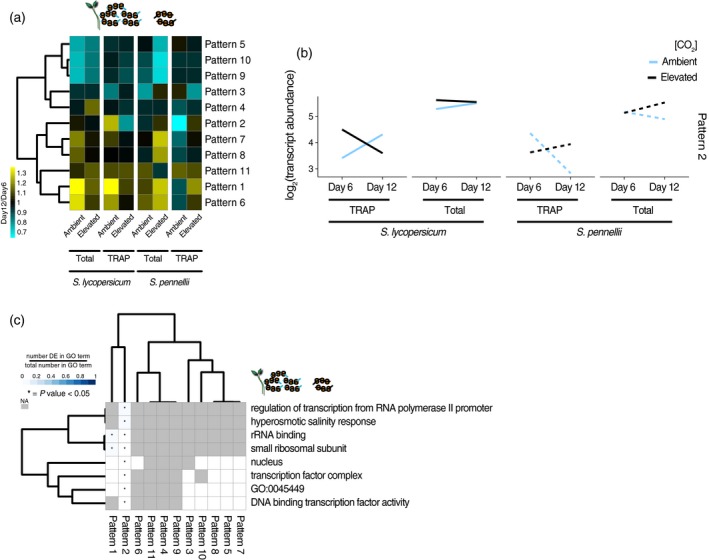
Genes with translational profiles that respond to eCO_2_ in a species‐specific manner are enriched in GO categories related to transcription. (a) Dominant patterns representing uniquely (upregulated or downregulated) differentially expressed genes that differ between total RNA and TRAP RNA. The colour scale represents temporal changes (12 DAP/6 DAP). (b) Genes that change expression between species in the ribosome‐associated RNA population. Lines represent the mean expression (as measured from transcript abundance in total RNA‐seq or ribosome loading in TRAP) of the genes (*n* = 140) assigned to Pattern 2. Blue lines = transcript abundance under ambient CO_2_; black lines = elevated CO_2_. Solid lines = *Solanum lycopersicum*, dashed lines = *S. pennellii*. (c) GO terms enriched in Pattern 2. Scale = ratio between the number of significant genes in the category and the size of the category. Asterisk = significant enrichment (GoSeq; *P* < 0.05). Cells in grey (NA) represent categories without enough genes to be considered in the analysis.

To test if changes in transcript–ribosome association occur in response to eCO_2_, we characterized these ribosome‐associated RNA populations in the shoots of both species at 6 and 12 DAP using Translating Ribosome Affinity Purification (TRAP)‐Seq from 35S:RPL18b‐His6‐FLAG‐GFP of *S. lycopersicum* cv M82 and *S. pennellii*. These time points were chosen as transcript accumulation related to translation initiation and elongation was enriched in the shoot and divergent at 12 DAP. In order to identify transcripts that are differentially loaded onto ribosomes (translational regulation), we selected 2599 upregulated or downregulated genes that showed differences in expression in response to eCO_2_ between both species in the transcriptome data or in the translatome data (Figure S5a,b; Experimental procedures). These 2599 genes could be further divided into 11 co‐regulated groups or ‘dominant patterns’ (Figure 5a). As an example, genes in Pattern 2 are decreased in transcript abundance in the ribosome‐associated fraction at 12 DAP in *S. lycopersicum* in response to eCO_2_, (while they typically increase over development time under ambient CO_2_) and show little to no difference in their abundance in the transcriptome data (Figure 5b). In contrast, these same genes in Pattern 2 are increased in their transcript abundance in response to eCO_2_ in *S. pennellii* in both ambient and in eCO_2_ (Figure 5b). To further characterize the biological processes associated with these differences between species in translationally regulated genes, we queried enriched GO categories (Figure 5c). GO terms related to transcriptional regulation and transcription factor activity were uniquely enriched in Pattern 2, a marked contrast with the translational regulatory pathways observed in the transcriptional profiling. Such enrichment for entire categories related to transcriptional regulation have not previously been identified in response to abiotic stresses (Mustroph *et al.*, 2009; Ribeiro *et al.*, 2012; Juntawong and Bailey‐Serres, 2012; Yángüez *et al.*, 2013; Aubry *et al.*, 2014; Lin *et al.*, 2014; Cheng *et al.*, 2015; Vragović *et al.*, 2015; Basbouss‐Serhal *et al.*, 2015; Zoschke *et al.*, 2016; Meteignier *et al.*, 2017; Xu *et al.*, 2017; Li *et al.*, 2018; Chen *et al.*, 2018; Wu *et al.*, 2019; Tian *et al.*, 2019). Other patterns had few and general terms associated with them (patterns 3, 6, 9, 10, and 11; Figure S6; Table S4).

The group of genes associated with Pattern 2 demonstrates feedback to transcriptional regulation in response to eCO_2_, which differs between species (Figure 5b and Table S4). Genes in this co‐regulated group comprise members of the Mediator complex (MED11, 18, and 19B), involved in transcription initiation (Seizl *et al.*, 2011; Buendía‐Monreal and Gillmor, 2016) and transcriptional repression (Dolan and Chapple, 2016), as well as 15 transcription factors. Subunits of the Mediator complex play roles in transcriptional regulation and in the abiotic stress response (Liao *et al.*, 2016). To the best of our knowledge, these have never been shown to be translationally regulated. Among the 15 transcription factors in this group, some have been associated with abscisic acid (ABA) and drought including Nuclear Factor Y (NF‐Y) (Warpeha *et al.*, 2007; Nelson *et al.*, 2007; Li *et al.*, 2008b), DEAR5/RAP2.9 (Agarwal *et al.*, 2006; Fujita *et al.*, 2011), and TCTP/P23 (Kim *et al.*, 2012). Together, these results suggested a translational regulatory mechanism in which ribosomal loading of transcripts associated with transcriptional initiation were selectively increased in *S. pennellii* and decreased in *S. lycopersicum*, thus providing transcriptional feedback. Similar to the transcriptome analyses, we anticipate a greater complexity of changes in translational abundance within and across individual cell types within both the shoot and the root.

**Figure 6:**
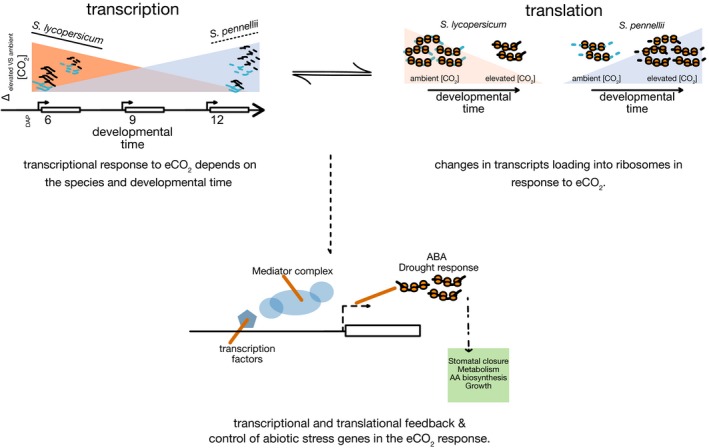
Model of the effects of eCO_2_ on transcription and translation in *Solanum* species. The transcriptional response to elevated CO_2_ has elements of species‐specificity, this depends on developmental stage and influences translation. A subset of genes is translationally regulated (Pattern 2, Figure 5b) in a species‐specific manner in response to elevated CO_2_. These genes include transcriptional regulators (members of the Mediator complex as well as other transcription factors) and genes associated with abscisic acid (ABA) response. We hypothesize that co‐ordinated feedback between transcriptional and translational regulation differs between these species, and yet gives rise to similar changes in metabolism, growth, and stomatal closure.

Overall, our results highlight the importance of a systematic understanding of the whole plant's response to eCO_2_ as well as the importance of molecular variation as a potential resource for breeding and can provide insight into the different means by which plant species can respond to elevated atmospheric CO_2_. Figure 6 offers an interpretation of how the molecular findings in this study underlie distinct global regulatory mechanisms that can lead to the same physiological response to eCO_2_. Our results point to the translational coordination of specific transcriptional machinery – the Mediator complex and other transcription factors – in a species‐specific manner. Further characterization of the potential adaptive role of development stage‐specific widening of xylem vessel diameter as well as of specific shoot and root metabolites identified here will be a valuable tool to help enable efforts to prepare for the upcoming increase in eCO_2_ and to sustainably prepare for the resulting changes to global agriculture.

## Experimental procedures

### Plants and growth conditions

Seeds of *S. lycopersicum* (cv M82) and *S. pennellii* (LA0716) were obtained from the C. Rick Tomato Genetics Resource Centre, sterilized, and sown in Profile Calcined Clay medium (MVP Products, Buffalo Grove, IL; www.turface.com) in 4‐inch pots with a modified removable bottom. Plants were grown in Conviron PGR15 growth chambers (Controlled Environments Ltd, Winnipeg, Manitoba, Canada; http://www.conviron.com). Chambers were set at either ambient (400 ppm) or eCO_2_ (700 ppm), 325 μmol m^−2^ sec^−1^ PPFD, 70% relative humidity, 16 h:8 h light:dark schedule, and 22°C at the Controlled Environment Facility at the University of California, Davis, USA. Carbon dioxide levels were monitored continuously with a monitor that immediately reported any major deviations in CO_2_ levels. No major deviations were recorded over the time period of the experiment. Plants were watered every 2–3 days with 40% Long‐Ashton solution containing 6 mm NH_4_NO_3_. The location of the flats within each chamber and the location of plants within each flat were rotated upon every watering, and CO_2_ treatment assignments were rotated between chambers at every experimental replication to avoid any experimental artefacts caused by differences between chambers.

Shoots and roots were harvested 28–35 DAP for shoot and root biomass. Twenty‐four plants (24 biological replicates) were measured for M82 and *S. pennellii*, respectively, for elevated and ambient carbon dioxide treatments. Shoots and roots were oven dried (at least 70°C), and dry biomass was recorded. Biomass measurements and the associated scripts to analyze these data can be found online at https://github.com/rodriguezmDNA/Gray_RodriguezMedina_CO2Supplemental.

### Measurement of photosynthetic rate and stomatal conductance

*In situ* leaf‐level photosynthetic rate (A) and stomatal conductance (g_sc_) were measured using the LiCOR 6400 system (LI‐COR; https://www.licor.com). Raw LiCOR measurements can be found at https://github.com/rodriguezmDNA/Gray_RodriguezMedina_CO2Supplemental. Measurements were taken consecutively over 3 days for *S. lycopersicum* and *S. pennellii* plants under ambient or eCO_2_ conditions in the youngest fully expanded leaf in 12‐week‐old plants. Measurements were taken from this leaf for three to five biological replicates per CO_2_ condition (ambient or elevated) for each species. The *anova()* function from *R* package *lmerTest* (R Core Team, 2013) (Kuznetsova *et al.*, 2017) was used to test the main effects of CO_2_ and species and their interaction in a linear model, treating the date of measurement as a random effect (Experimental procedures). The experiments were repeated four times. Measurements were taken between 15:00 and 18:00, consecutively over 3 days.

### Measurement of root anatomy and morphology

Whole root systems were collected at 7, 10, and 13 DAP, submerged in deionized water in a shallow, clear dish, and scanned on a flatbed scanner (Epson Perfection V700 Photo Scanner; Epson America, Long Beach, CA, USA; https://epson.com/usa). Images were analyzed using the ImageJ plugin SmartRoot (Lobet *et al.*, 2011) to provide data on total root system length, number of lateral roots, and root diameter. Following scanning, 1 cm samples were collected at 2–3 cm from the root tip, embedded in agarose and preserved in FAA for later cross‐sections. A linear model in *R* (R Core Team, 2013) was used to test for significant main effects of CO_2_, species, and developmental time, and their two‐way and three‐way interactions.

### Cellular anatomy measurements of roots

Root tissue samples that had been preserved in FAA were rehydrated using a stepwise series of ethanol concentrations and sectioned into 200‐µm thick sections using a Vibratome Series 1000 sectioning system. Sections were stained with toluidine blue, de‐stained in water, and mounted in 50% glycerol. Sections were imaged using an Olympus Vanox microscope (www.olympus-lifescience.com), and analyzed using ImageJ for cross‐sectional diameter, and diameter of the largest metaxylem vessel. Cross‐sections were prepared, imaged, and analyzed from root tissue collected from *S. lycopersicum* and *S. pennellii* plants at four time points: 7, 10, 13 DAP, and at 12 weeks old. Raw data for root anatomical measurements can be found at https://github.com/rodriguezmDNA/Gray_RodriguezMedina_CO2Supplemental. Four biological replicates were analyzed for each time‐point (7, 10, and 13 DAP) in either ambient or elevated carbon dioxide in each species. When measuring xylem vessel diameter in mature roots, 11–12 biological replicates (representing sections from different plants) were analyzed from lateral roots and primary roots, for each carbon dioxide concentration and for each species. Only xylem vessels larger than 20 µm in diameter were considered, following the method in Rincon *et al.* (2003). A linear model in R was used to test for significant main effects of CO_2_, species, and developmental time, and their two‐way and three‐way interactions. Outliers were either substituted by their group average between replicates (for young xylem measurements) or removed (for mature metaxylem measurements).

### Tissue collection for RNA‐seq and metabolite profiling

Whole root and whole shoot samples were collected for transcriptomic and metabolomic profiling at 6, 9, and 12 DAP. To collect each sample, the entire pot was lowered into deionized water and then the pot was slowly lifted up and away from the removable bottom of the pot, allowing the medium to gently float away from the root system. The plant was then moved to a secondary container of deionized water to ensure that the roots were free of medium. Then, the roots and shoots were severed using a clean razor blade, and the tissue was flash‐frozen in liquid nitrogen. From submergence of the root system to placement of samples in liquid nitrogen, a maximum of *c.* 75 sec elapsed.

For transcriptome profiling, root tissue from three individual plants was pooled together to yield one root sample per treatment‐species combination day^–1^ on each of the three sampling days, and shoot tissue from the same three plants was pooled together to yield one shoot sample per treatment‐species combination day^–1^ on each of the three sampling days. The same sampling scheme and chambers were used for metabolite and physiological profiling. The experiments were repeated four times for all traits measured.

### RNA‐seq library preparation

RNA‐seq libraries were prepared using the method described by Kumar *et al.* (2012). Briefly, for each library, the pooled root or shoot samples were homogenized in lysis and binding buffer containing B‐mercaptoethanol. Tissue was disrupted using a mortar and pestle and a Mini‐beadbeater‐96 high‐throughput cell disruptor (BioSpec Products, Bartlesville, OK, USA; www.biospec.com). Oligo(dT)_25_‐coated Dynabeads (Ambion, Foster City, CA, USA; www.thermofisher.com) were then added to the lysate, and mixed for 10 min to allow oligo(dT) beads to hybridize to poly‐adenylated mRNA. cDNA was then synthesized and digested to *c. *300 bp fragments using NEBNext DNA fragmentase enzyme mix (NEB, Beverly, MA, USA; www.neb.com). Library fragments greater than 300 bp were purified using AMPure XP solid phase reverse immobilization (SPRI) magnetic beads (Agencourt Bioscience, Beverly, MA, USA). For each library, one of the 96 unique barcoded adapters described in Kumar *et al.* (2012) was ligated to each library, enabling multiplexing. cDNA was quantified using a SYBR green‐based method on a plate reader, libraries were pooled, and fragment size and quality was checked using a BioAnalyzer (Agilent Technologies, Santa Clara, CA, USA; www.agilent.com).

### Translating ribosome affinity purification

To determine the effects of eCO_2_ on the translatome of shoot tissue, transgenic *S. lycopersicum* and *S. pennellii* expressing a FLAG epitope on the ribosomal L18 (RPL18) under the 35S promoter (Ron *et al.*, 2013) were grown in ambient and elevated CO_2_ under the same conditions and chambers as described above. At 6 and 12 DAP, tissue was collected for the TRAP protocol. Because the biomass of plant tissues in young seedlings was small, and because the TRAP populations were segregating, we pooled multiple plants (up to 15) together to ensure that adequate mRNA for library preparation could be collected via the ribosomal pulldown.

The TRAP procedure has been described previously by Zanetti *et al.* (2005). Briefly, tissue from transgenic plants expressing the FLAG epitope was ground in liquid nitrogen using a mortar and pestle, and homogenized in polysome extraction buffer. Samples were clarified by centrifugation and filtration through a Miracloth, and the clarified extract was added to magnetic beads that were coated in anti‐Flag antibody. Beads and extract were incubated at 4°C using a nutator for 2 h, and washed six or seven times with wash buffer. Polysomes bound to magnetic beads were then frozen at −80°C until RNA‐seq library preparation.

RNA‐seq libraries were prepared from TRAP polysomes using the method described by Townsley *et al.* (2015). Lysis and binding buffers containing B‐mercaptoethanol were added to anti‐Flag magnetic beads and bound ribosomes, allowing elution of mRNA. mRNA was then hybridized to biotin–20nt‐dT oligos, allowing pulldown of mRNA out of solution with the use of magnetic streptavidin beads. mRNA was then washed with three successive buffers, eluted off the beads at 80°C, and a secondary wash was performed by adding more biotin–20nt‐dT oligos to the mRNA, repeating the bead pulldown and the buffer washes. mRNA was then heat fragmented, and first and second strand cDNA was synthesized. The optimal fragment size was selected using AMPure beads, and libraries were enriched using PCR.

### Library sequencing and bioinformatics pipeline

Pooled and barcoded libraries were submitted to the Vincent J. Coates Genomic Sequencing Facility at UC Berkeley (http://qb3.berkeley.edu/qb3/gsl/index.cfm) and were sequenced on an Illumina HiSeq 2000 instrument (https://www.illumina.com). A custom Perl script was used to split sequence reads into individual libraries based on barcode sequences (Toal *et al.*, 2018), and FastQC (Andrews, 2010) was used to plot read quality. We used the bowtie 1.2.2 tool (Langmead *et al.*, 2009) to align RNA‐seq reads to the *S. lycopersicum* reference transcriptome (ITAG 3.2, http://www.solgenomics.net). Mapped reads were aggregated into a counts table using a custom R script and used in downstream expression and clustering analysis. Raw total and TRAP sequencing data can be obtained from the Gene Expression Omnibus (GEO) under the accession number http://www.ncbi.nlm.nih.gov/geo/query/acc.cgi?acc=GSE126430. Normalized CPM data are found in Table S5.

### Differential expression analysis

Counts were normalized using edgeR (Robinson *et al.*, 2009) and limma‐voom (Ritchie *et al.*, 2015) Bioconductor packages in R. A linear model of the main and interaction effects between CO_2_, species, and developmental time was used to test for differentially expressed genes. Data from roots or shoots were analyzed separately. False discovery rates were calculated using the ashR (Stephens, 2016) package in R. Normalized CPM data for total and TRAP RNA‐Seq is available in Table S5. Correlation between all samples is also in Table S5. Only samples present in these tables were considered for the analyses.

### Clustering analysis

Voom‐normalized counts were calculated using the *voom()* function from the limma package. These values were used for clustering analysis, and selecting for the top varying genes. This analysis was carried out using the algorithm described by Orlando *et al.* (2009). Genes assigned to each dominant pattern are listed in Table S4.

### Functional enrichment analysis

Enrichment of GO categories was analyzed using GOseq (Young *et al.*, 2010). For custom gene lists, Fisher's Exact Tests were used to test for enrichment. The glycolysis and TCA cycle‐related genes that are eCO_2_‐responsive were obtained from the list described in Markelz *et al*. (2014a). Multiple hypothesis testing was not performed for GO categories due to the graph structure of GO terms. It has been discussed that enrichment tests for different GO categories can be correlated, and therefore multiple testing corrections in GO analyses cannot be simply addressed by calculating ordinary false discovery rates (Mi *et al.*, 2012) or that the correction can be too conservative and yield few or no significant terms (Alexa and Rahnenfuhrer, 2007).

### Motif enrichment

We used AME (McLeay and Bailey, 2010) to identify enriched transcription factor binding motifs in the 1 kb region upstream from the transcription start site of genes assigned to dominant patterns 3, 5, 9, 10, and 11. The parameters were set as follows: ‐‐verbose 1 ‐‐scoring avg ‐‐method fisher ‐‐hit‐lo‐fraction 0.25 ‐‐evalue‐report‐threshold 10.0 ‐‐control ‐‐shuffle‐‐ ‐‐kmer 2. The Arabidopsis DAP‐Seq (O'Malley *et al.*, 2016) database was used as a reference.

### Untargeted metabolite profiling

Tissues were collected for metabolite profiling at the same time and using the same method as the tissue collection for the RNA‐seq experiment. Root and shoot tissue were separately pooled for three individuals of each species and each CO_2_ treatment at 6, 9, or 12 DAP (four biological replicates each). Samples were lyophilized, ground, and extracted in a chilled mixture of methanol, chloroform, and water (5:2:2). Samples were then vortexed, centrifuged, and the supernatant was dried (Labconco Centrivap cold trap concentrator), and resuspended in degassed acetonitrile and water (1:1) to clean the sample of membrane lipids and triglycerides. Samples were again dried, Fatty Acid Methyl Esters (FAMEs) of eight to 30 carbons in length were added as internal standards, and samples were then derivatized using methoxyamine hydrochloride in pyridine followed by *N*‐methyl‐*N*‐(trimethylsilyl)trifluoroacetamide. Data were acquired using gas chromatography and time‐of‐flight mass spectrometry as described by Fiehn *et al.* (2008). Briefly, a volume of 0.50 μl was injected for each sample on an rtx5Sil‐MS column (Restek Corporation; Bellefonte, PA). The temperature gradient was as follows: 50°C for 1 min, increasing 20°C min^−1^ up to 330°C, and held constant at 330°C for 5 min. Compound identification was accomplished via mass spectrometry (Leco Pegasus IV mass spectrometer; Leco Corporation, St. Joseph, MI). Here, 191 compounds were identified in the samples from this experiment. Sample extraction, derivatization, and data acquisition were all completed at the West Coast Metabolomics Facility (http://metabolomics.ucdavis.edu/). Data for each metabolite were first log_2_ transformed and then a linear model was fit to test for main effects of CO_2_, species and developmental time, and their interactions using the *R* function *lm()*. Multiple testing correction was performed using the ashR package in R. Roots were analyzed separately from shoots. Log_2_ transformed data and results from the linear model and *P*‐value correction are in Table S3. Data can also be found at https://github.com/rodriguezmDNA/Gray_RodriguezMedina_CO2Supplemental.

### Selection of a gene set with unique transcriptional and translational profiles

To select uniquely upregulated or downregulated genes at the transcriptional or translational level, we performed pairwise comparisons to test for the effect of CO_2_ between samples of the total or TRAP, species (*S. lycopersicum* or *S. pennellii*), and day (6 or 12 DAP), with eight contrasts. Differentially expressed genes (*q*‐value < 0.01) were separated on the basis of their direction of change: upregulated (logFC > 1) or downregulated (logFC < 1). For each list of upregulated or downregulated genes, we performed a set analysis between samples of the same RNA source on both days (6 and 12 DAP) and species. To identify genes uniquely regulated in each species, we took the symmetric difference between species regardless of development time response.

## Conflict of interest

The authors declare no affiliations with or involvement in any organization or entity with any financial interest, or non‐financial interest in the subject matter or materials discussed in this manuscript.

## Author contributions

SBG and SMB conceived the original research and experiment design; SBG performed the experiments; SBG and JRM performed analysis of the data.; SBG, JRM, and SMB interpreted the results and wrote the article. KK performed TRAP sequencing experiments. SR and TWT provided technical assistance to SBG; DR provided significant guidance on data analysis. SMB agrees to serve as the author responsible for contact and ensures communication.

## Supporting information


**Figure S1.** Whole root to metaxylem ratios.
**Figure S2.** Elevated CO_2_ has no significant effects on mature metaxylem in either species.
**Figure S3.** Number of significantly differentially expressed genes in each of the linear model terms in shoots or roots.
**Figure S4.** Clustering of genes that change in a (CO_2_ × Species × Day 12) interaction manner reveals genetic modules with dominant patterns.
**Figure S5.** Strategy to identify groups of genes with unique transcriptional or translational patterns.
**Figure S6.** Number of GO terms enriched per dominant pattern in total and TRAP RNA‐Seq data.

**Table S1.** anova tables from root area and diameter analyses

**Table S2.** anova tables from shoot or root biomass, photosynthesis, and stomatal conductance analyses

**Table S3.** Log_2_ transformed data and results of the metabolomic analysis

**Table S4.** Genes assigned to dominant patterns and motif enrichment analysis

**Table S5.** CPM normalized data from total and TRAP RNA‐Seq

## Data Availability

Raw total and TRAP sequencing data can be obtained from GEO under the accession number http://www.ncbi.nlm.nih.gov/geo/query/acc.cgi?acc=GSE126430. Normalized CPM data for total and TRAP RNA‐Seq are provided in Table S5. Raw LiCOR, root anatomy and morphology and associated scripts can be found at https://github.com/rodriguezmDNA/Gray_RodriguezMedina_CO2Supplemental.
