# Supplementary material for: Translational regulation contributes to the elevated CO2 response in two Solanum species
Source: Plant J. 2020 Jan 16;102(2):383–97. doi: 10.1111/tpj.14632 (PMC7216843; doi:10.1111/tpj.14632)
Supplement: Supplementary file 1 — Figure S1. Whole root to metaxylem ratios. Figure S2. Elevated CO2 has no significant effects on mature metaxylem in either species. Figure S3. Number of significantly differentially expressed genes in each of the linear model terms in shoots or roots. Figure S4. Clustering of genes that change in a (CO2 × Species × Day 12) interaction manner reveals genetic modules with dominant patterns. Figure S5. Strategy to identify groups of genes with unique transcriptional or translational patterns. Figure S6. Number of GO terms enriched per dominant pattern in total and TRAP RNA‐Seq data. [file TPJ-102-383-s001.pdf]

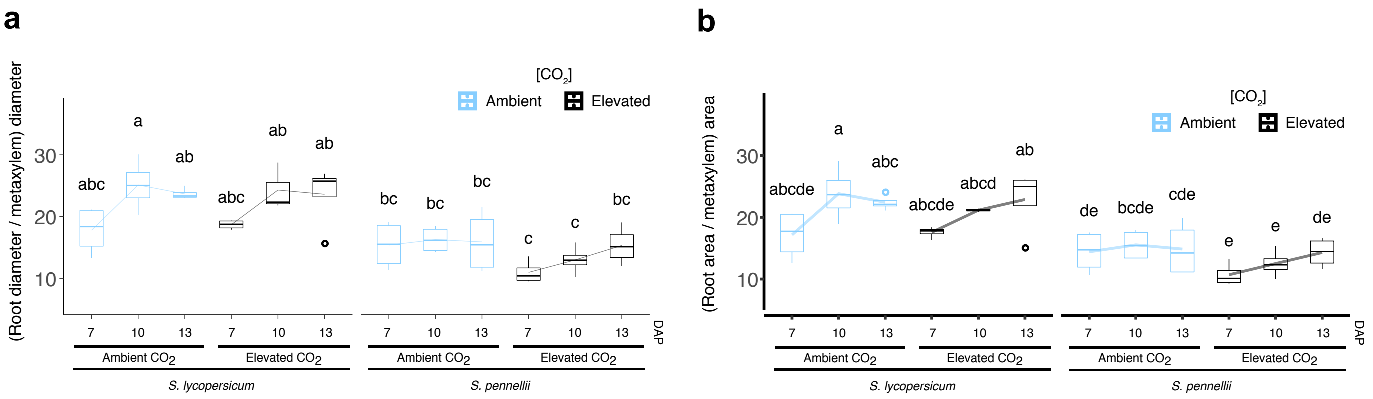

Figure S1. Whole root to metaxylem ratios. (a,b) represent the ratio of whole root to metaxylem changes for diameter **(a)** or area **(b)**. Letters above the boxplot are the results from a Tukey HSD test.

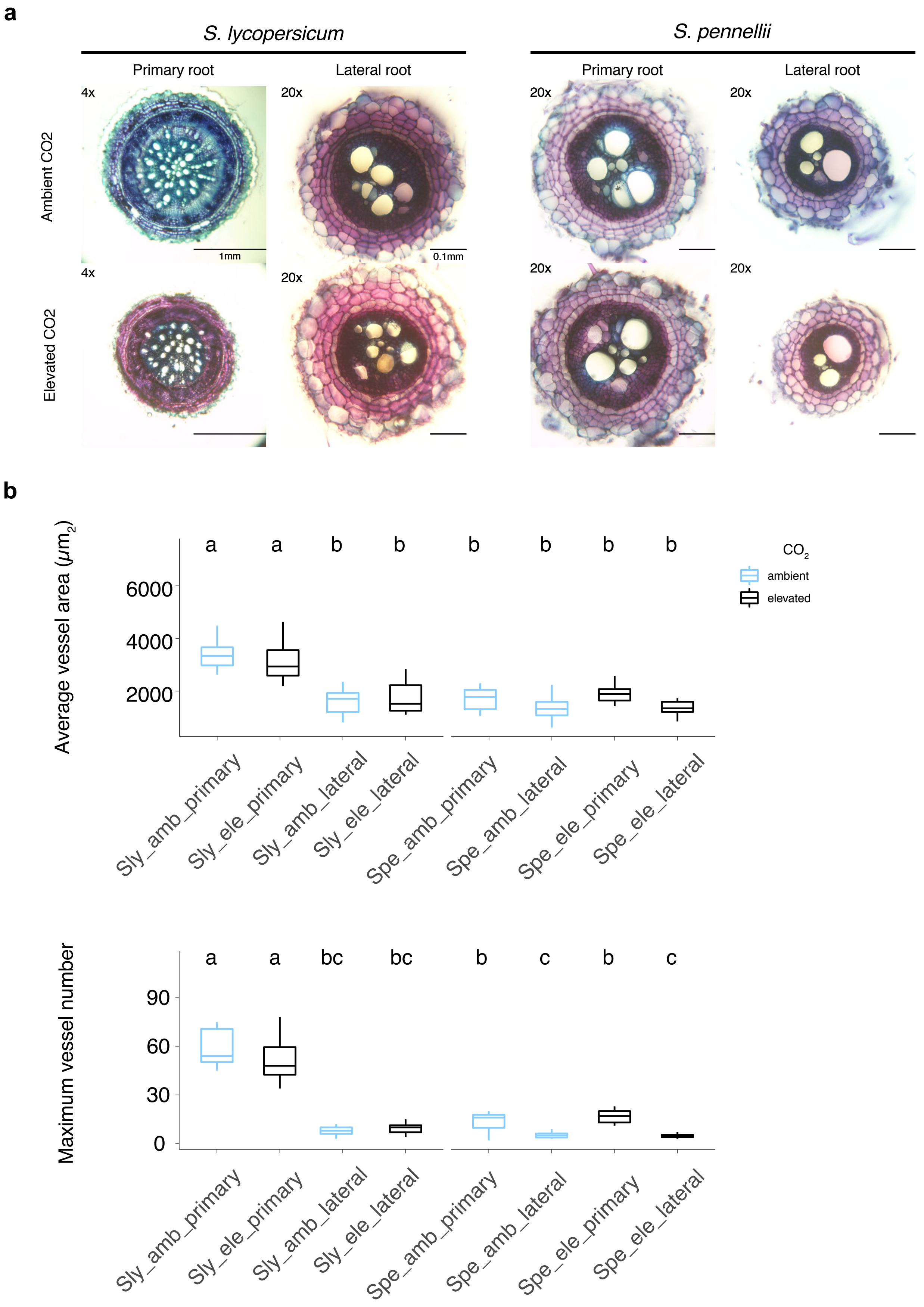

Figure S2. Elevated CO_2 has no significant effects on mature metaxylem in either species. (**a**). Cross sections from roots taken at 1cm from the tip of lateral or primary roots from *S. lycopersicum* cv. M82 (left half) or *S. pennellii* (right half) at 12 weeks after planting, under ambient (upper row) or elevated (bottom row) CO_2. (**b**) Distribution of the average vessel area (upper panel) and number of vessels (bottom panel). CO_2 had no significant effect ($p > 0.05$) on the average area of lateral or primary roots in a 2-way ANOVA (~CO_2 X Species); number of vessels wasn't significantly affected ($p > 0.05$) by CO_2 in a 3-way ANOVA (~CO_2 X Species X Root order); root order = primary or lateral roots (**Table S2**).

a

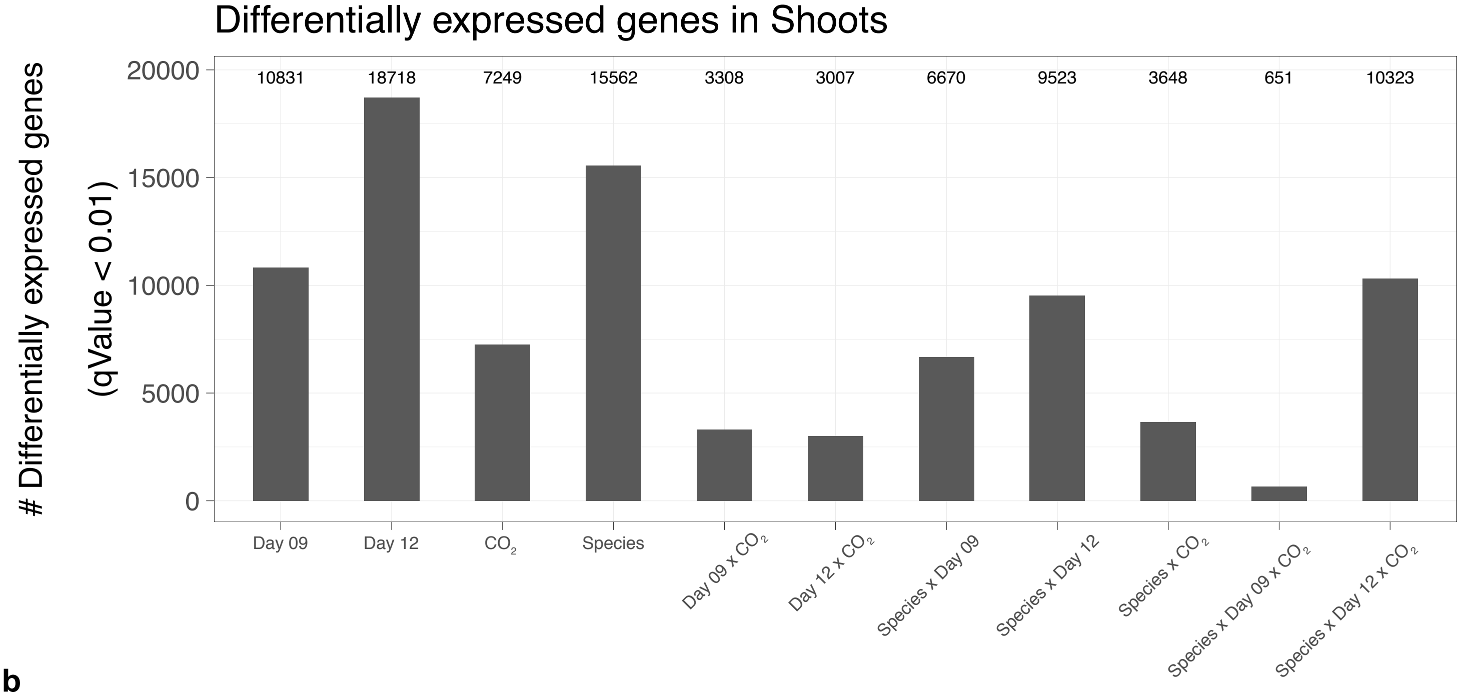

b

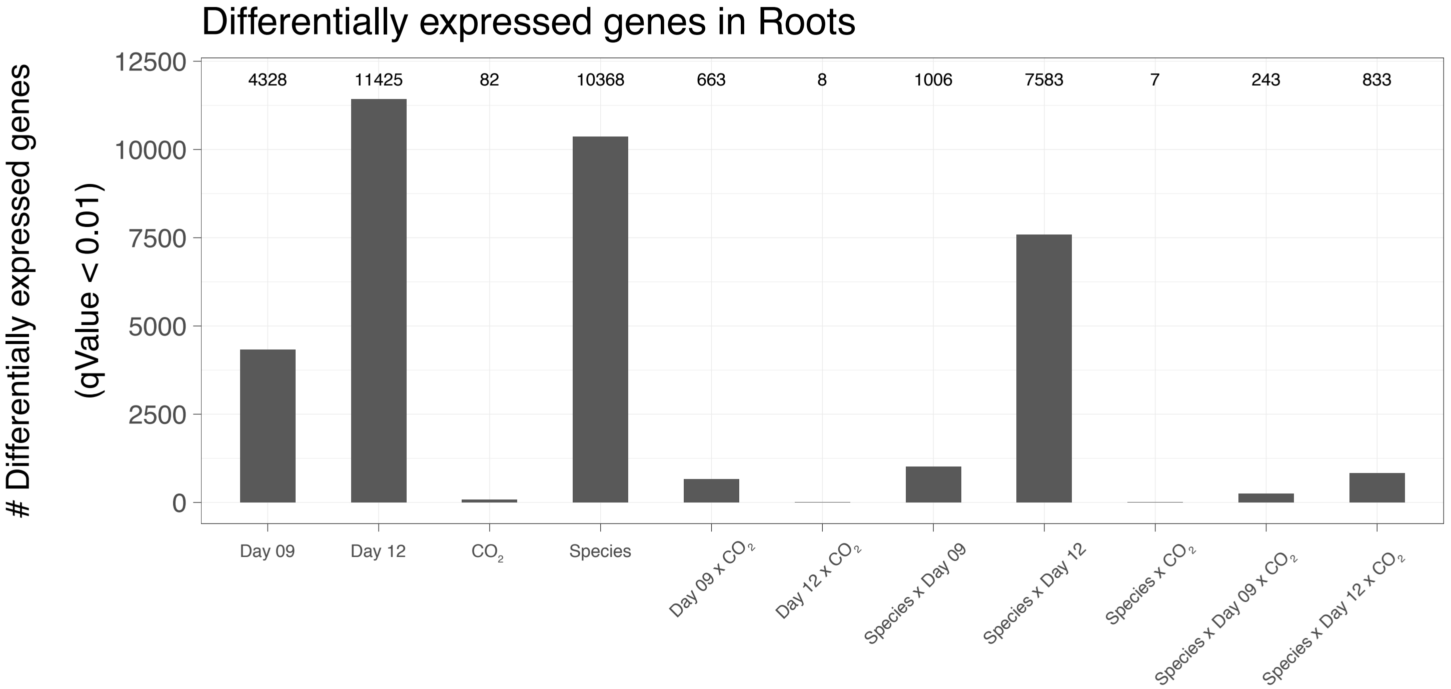

Figure S3. Number of significantly differentially expressed genes in each of the linear model terms in shoots or roots. The barplots show the number (y-axis) of significant differentially expressed genes (q-value < 0.01) in each of the terms (x-axis) in the linear model. Changes in expression were measured using a linear model. The linear model coefficients include the main effects of [CO_2] (ambient or elevated), species (*S. lycopersicum* or *S. pennellii*) or developmental-time (days 6, 9 or 12) as well as the 2-way and 3-way interactions. Figures show the results for the changes in expression in shoots (**a**) or roots (**b**).

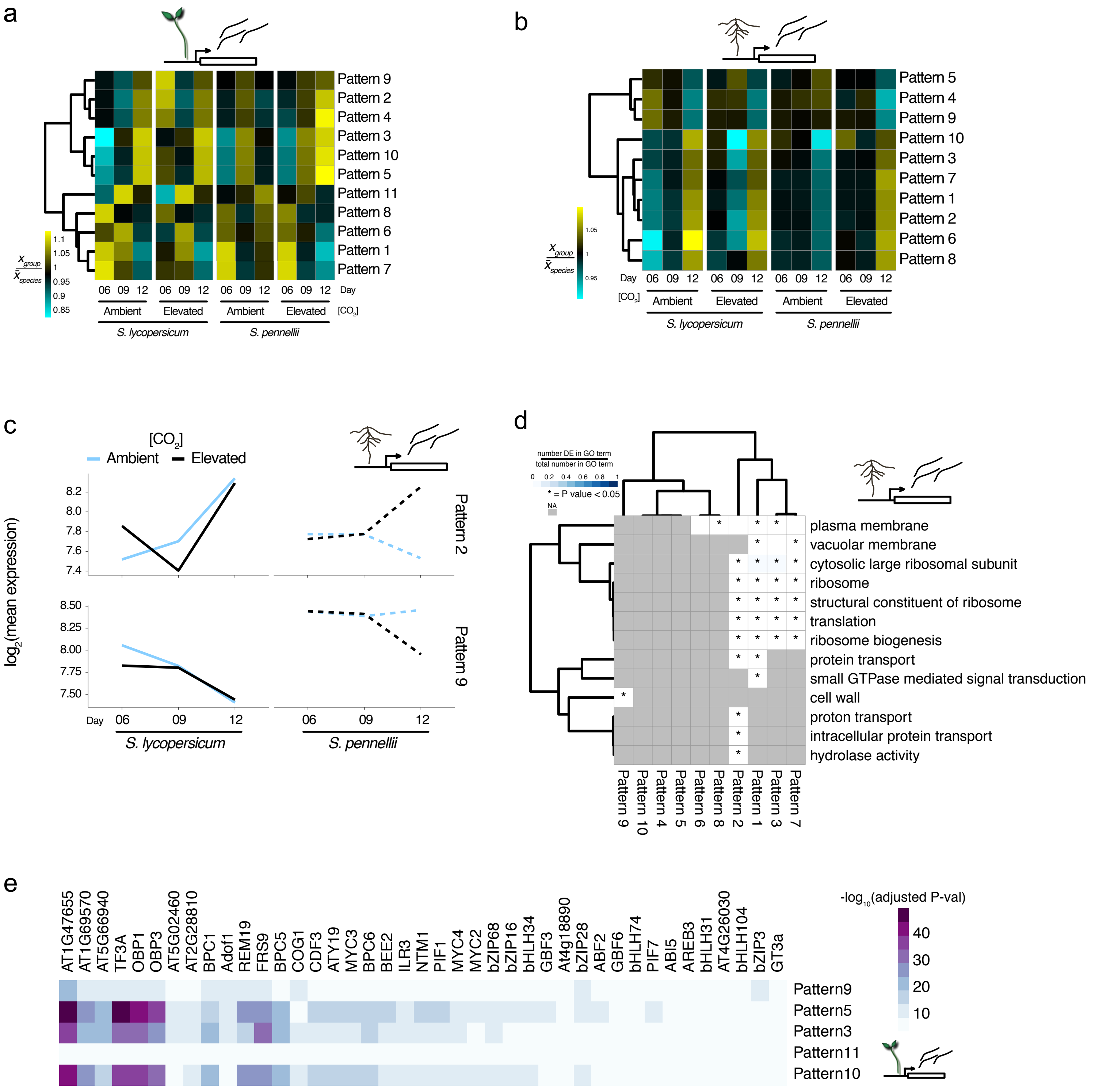

Figure S4. Clustering of genes that change in a (CO_2 X Species X Day 12) interaction manner reveals genetic modules with dominant patterns. (a,b) shows the row-wise scaled expression (expression of the group/mean expression by species) of groups of genes assigned to dominant patterns of expression (**Methods**) in shoots **(a)** or roots **(b)**. **(c)** Representative line plots of root dominant patterns in (**b**) are an alternate visualization of the heatmap in (**b**). Lines represent the average log2 normalized expression of all genes assigned to a dominant pattern; blue represents expression under ambient CO_2 (400 ppm); black, expression under elevated CO_2 (700 ppm). (**d**) Heatmap of enriched GO categories for root clusers. The scale shows the ratio between the number of significant genes in the category and the total number of genes assigned to the same category. An asterisk indicates if the category is significantly enriched ($p < 0.05$) in that cluster. (**e**) Enriched motifs common to shoot genes in patterns 3,5,9,10, and 11 (Scale shows the -log10 of the adjusted P value). Results from enrichment analysis are in **Table S4**.

a

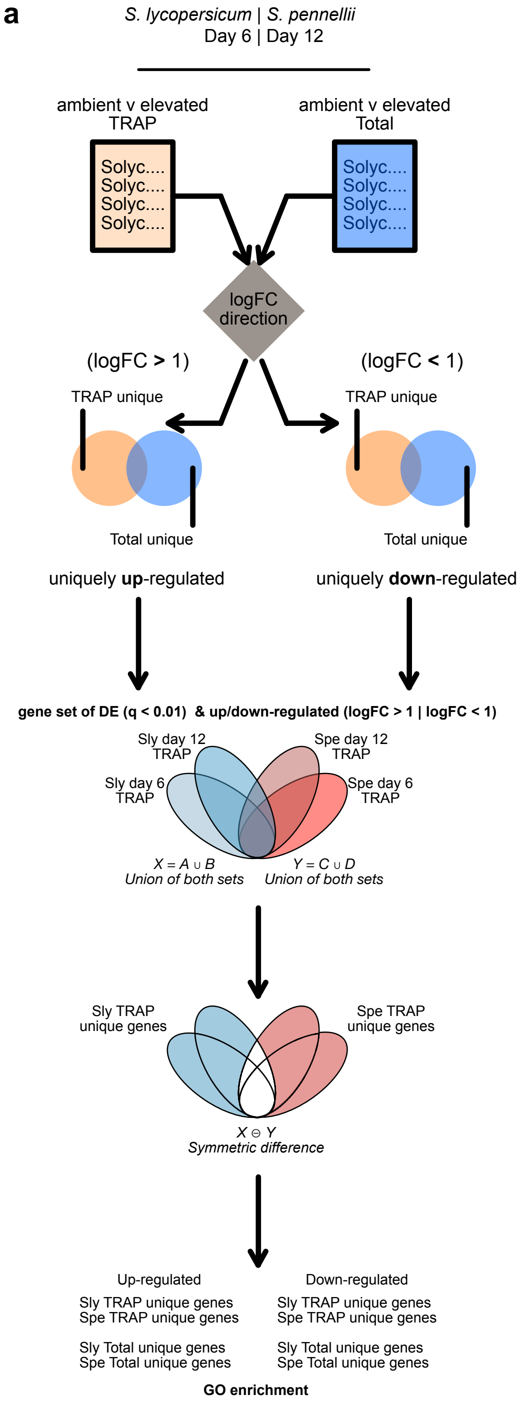

Figure S5. Strategy to identify groups of genes with unique transcriptional or translational patterns. (**a**) illustrates the process followed to identify genes that were uniquely regulated at the translational level. We tested for pairwise comparisons between ambient and elevated [CO_2] at 6 or 12 days in each species and RNA source (total or TRAP) independently. A total of 8 comparisons were made. Genes that were significantly differentially expressed (q-value < 0.01) were filtered by direction of change (up or down regulated). Comparable samples of total or TRAP (same day and species contrast of ambient versus elevated CO_2, but different RNA source) were compared to obtain unique sets of genes being up or down-regulated in TRAP or total (ignoring the overlap). In total, we obtained 16 lists of genes changing in either direction (up or down-regulated) unique to TRAP or total RNA. We further compared these 16 sets to select genes unique to each specific set and filter out genes shared with any other set. (**b**) A total of 2,599 unique genes were considered for downstream analysis.

b

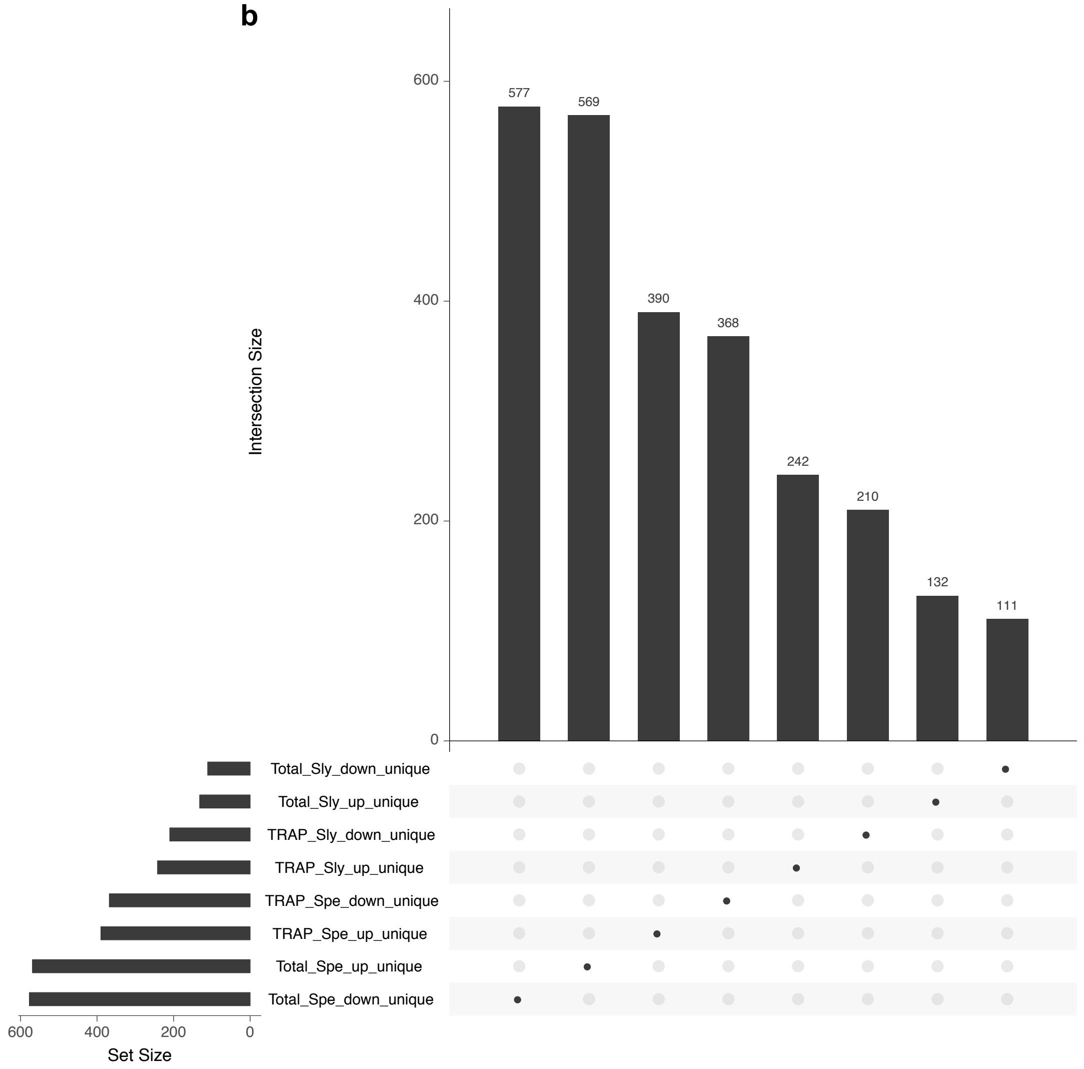

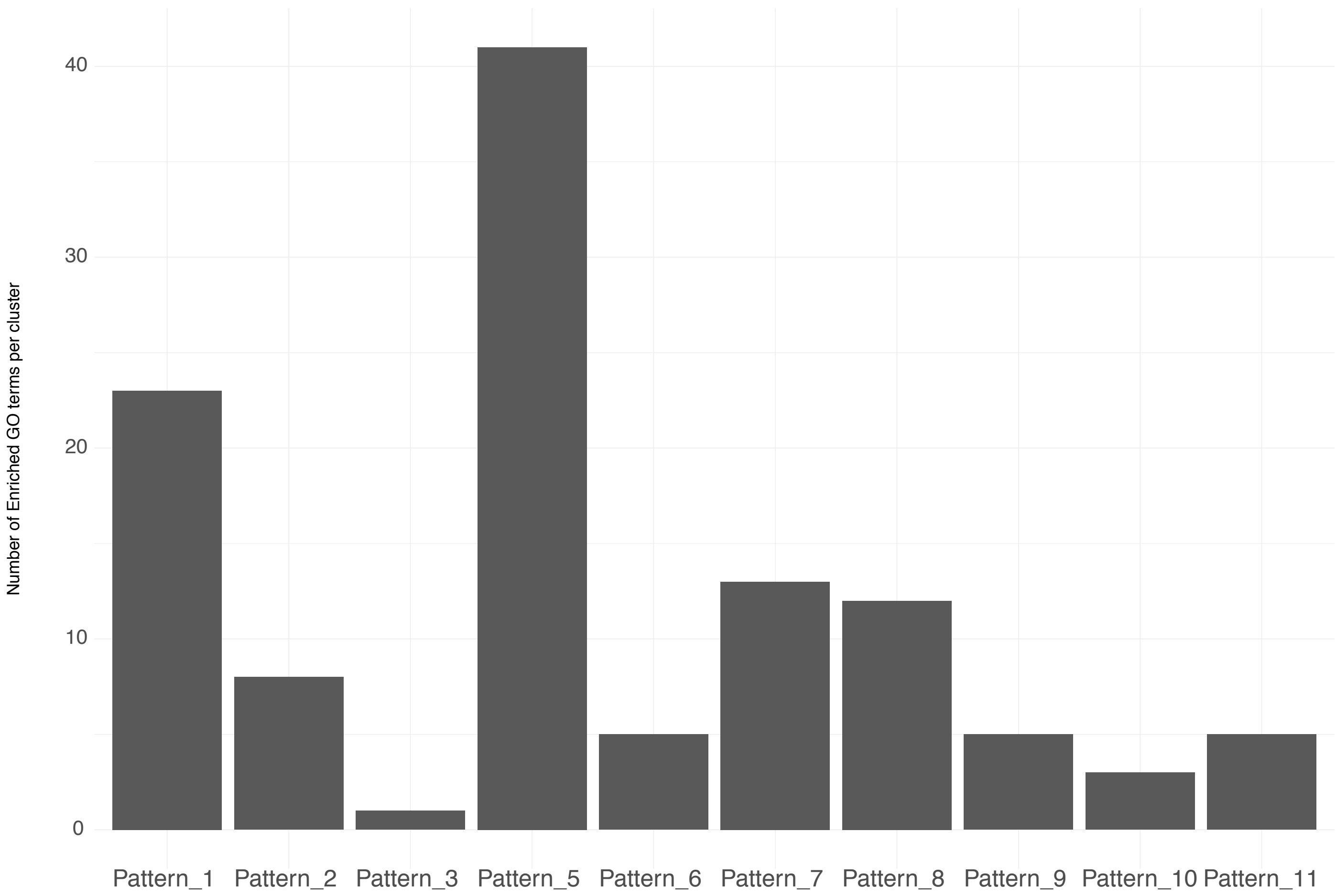

Figure S6. Number of GO terms enriched per dominant pattern in total and TRAP RNASeq data. Number of enriched GO terms for genes assigned to dominant patterns (**Figure 5**) using the Dominant Pattern finding algorithm (**Methods**). Only significant GO terms with >= 3 differentially expressed genes were considered (**Table S4)**.
